# A Rare Case of Anti-glycyl transfer RNA (tRNA) Synthetase Antibody-Related Non-specific Interstitial Pneumonia

**DOI:** 10.7759/cureus.26159

**Published:** 2022-06-21

**Authors:** Ram Chandra Khatri Chhetri, Shrey Gole, Arvin Junn P Mallari, Aman Dutta, Farah Zahra

**Affiliations:** 1 Internal Medicine, Chicago Medical School Internal Medicine Residency Program at Northwestern McHenry Hospital, McHenry, USA; 2 Internal Medicine, Chicago Medical School Internal Medicine Residency Program at Northwestern Medicine McHenry Hospital, McHenry, USA

**Keywords:** anti ars antibody, myositis specific antibody, anti-glycyl t-rna synthetase, non-specific interstitial pneumonia, anti-ej antibody, anti-synthetase syndrome

## Abstract

This is a case of a 65-year-old female with a past medical history of type 2 diabetes mellitus (DM) and hypothyroidism who presented with a five-day history of shortness of breath, dry cough, and fatigue. Shortness of breath was exertional, and cough was intermittent. She had no exposure to COVID-19 infection. During the presentation, the patient required supplemental oxygen up to 6 liters per minute (L/m) and was tachypneic and tachycardic. Initial computed tomography (CT) of the chest revealed bilateral parenchymal disease compatible with COVID-19 pneumonia, however, the patient's COVID-19 polymerase chain reaction (PCR) test was persistently negative. Despite being treated for COVID-19 pneumonia, the patients’ oxygen requirement increased, leading to the requirement of non-invasive positive pressure ventilation (BiPAP - bilevel positive airway pressure). The pulmonologist initiated a workup for possible underlying interstitial lung disease (ILD). Anti-glycyl transfer RNA (anti-EJ) antibody was positive on two occasions. The patient was started on pulse dose steroid and long-term steroid taper. The patient responded very well to the steroid and was later able to wean off the oxygen to room air. High-resolution CT which was done 3 months after the hospital stay revealed features suggestive of non-specific interstitial pneumonia (NSIP). Anti-synthetase syndrome is a rare but treatable etiology of ILD and should always be considered as a differential during workups.

## Introduction

Anti-glycyl transfer RNA (anti-EJ) antibody is one of the many anti-aminoacyl-tRNA synthetase (anti-ARS) antibodies that target the enzymes that catalyze the binding of amino acids to their respective transfer RNA (tRNA) during protein synthesis. To date, eleven anti-ARS antibodies have been identified: anti-Jo-1, anti-PL-7, anti-PL-12, anti-EJ, anti-OJ, anti-KS, anti-Zo, anti-Ha, anti-JS, anti-SC, and anti-YRS [1]. Nonspecific interstitial pneumonia (NSIP) is one of the various types of interstitial lung diseases (ILDs), which can be associated with connective tissue diseases (CTD), drugs, hypersensitivity pneumonia, but are usually idiopathic. However, despite failing to meet the specific diagnostic criteria for CTD, a large subset of ILDs have clinical features that suggest an autoimmune process. These subsets have been classified as interstitial pneumonia with autoimmune features (IPAF) [2]. Anti-ARS syndrome is a syndrome characterized by ILD, myositis, arthritis, Raynaud’s phenomenon, and mechanic’s hand [3]. The clinical features and outcomes vary with each type of Anti-ARS antibody [4]. ILD is one of the commonest clinical manifestations of the anti-ARS syndrome, and its prevalence ranges from 67-100% [5]. NSIP manifests with dyspnea and cough, and imaging studies like high resolution computed tomography(HRCT) show increased reticular markings, traction bronchiectasis, lobar volume loss, and ground-glass opacity. 

In patients who are positive for anti-ARS antibody, the clinical manifestations of ILD are not well established. However, NSIP has been reported in patients with anti-Jo-1, anti-PL-7, and anti-EJ antibodies [6]. The present manuscript presents the clinical, laboratory, and radiological findings of a patient with anti-EJ antibodies to add to the current knowledge regarding the possible presentations of this syndrome.

## Case presentation

A 65-year-old female with a past medical history of insulin-dependent type II diabetes mellitus (DM) and hypothyroidism presented with complaints of five days of worsening dyspnea on exertion, nonproductive cough, and fatigue. Her weight was 210 pounds. She did not experience fever, chest pain, loss of taste, urinary symptoms, or exposure to COVID-19. However, she was not vaccinated for COVID-19. She was a former smoker, having quit 20 years prior, but denied using alcohol or other recreational drugs. She did not have any exposure to pets, birds, natural gas, or toxins. However, she reported frequently using an essential oil diffuser at home.

**Figure 1:**
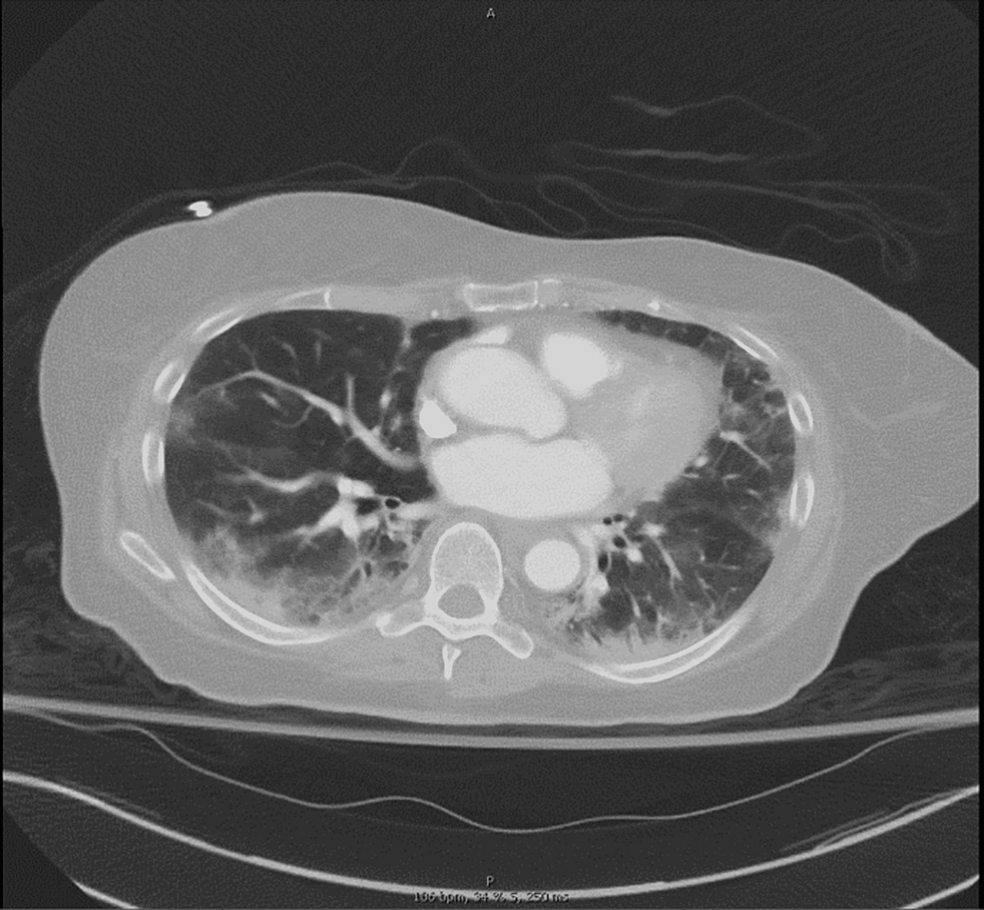
Diffuse parenchymal opacity in the lower lung fields with ground-glass opacities.

During the initial presentation in the emergency department, she was hypoxic with a saturation of 80% on room air, tachycardic (pulse 120 bpm), and tachypneic (respiratory rate: 28). Physical examination revealed bibasilar inspiratory crackles, without any other significant finding on exam. She was administered oxygen supplementation via a high flow nasal cannula at 6 L/m, maintaining saturation above 95%. Polymerase chain reaction for COVID-19 was negative. The patient was severely acidotic with a pH of 6.96, HCO3 (bicarbonate) of 4 mmol/L (21-32 mmol/L), and ketones in her urine, and was diagnosed with diabetic ketoacidosis (DKA). Chest computed tomography (CT) showed ground-glass opacities suggestive of COVID-19 pneumonia (Figure 1).

**Figure 2:**
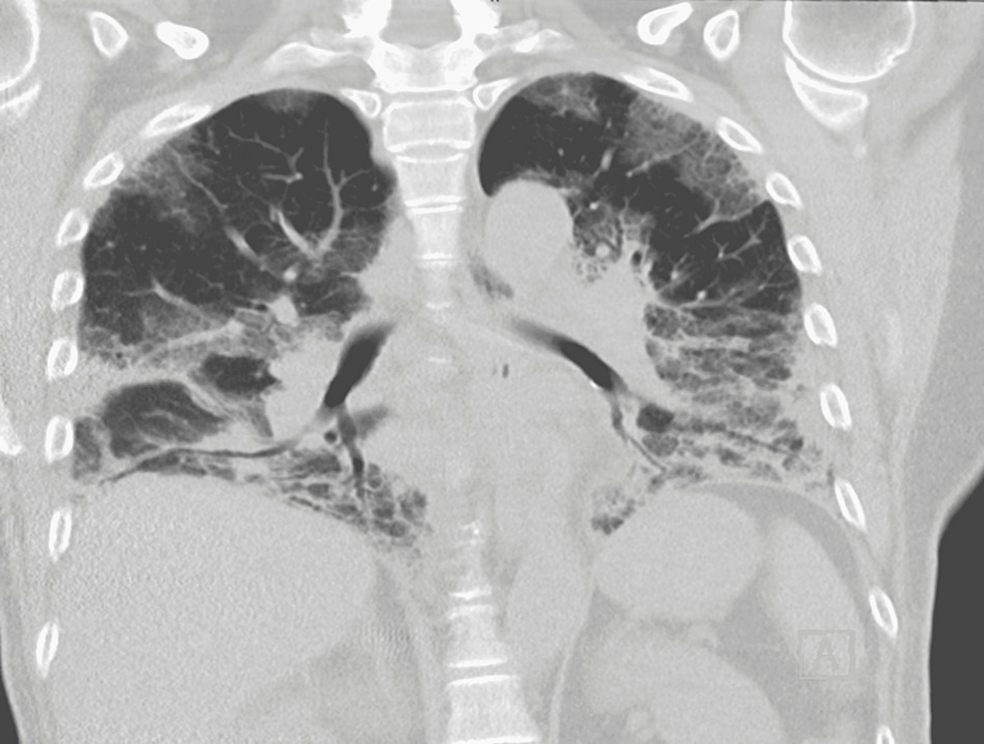
Extensive peripheral interstitial disease with end-stage bibasilar airspace consolidation.

The patient’s acidosis resolved after appropriate management of her DKA. She was admitted for seven days in the hospital. Her oxygen saturation was 93% on 3 L/m. She was discharged home with supplemental oxygen of 3 L/m. However, she returned to the hospital the following day with severe dyspnea. COVID-19 testing was again negative. Her oxygen supplementation requirements increased to 8 L/m, with the patient requiring BiPAP (bilevel positive airway pressure) for adequate oxygenation. The patient was evaluated for possible ILD, which showed elevated erythrocyte sedimentation rate (ESR), elevated C-reactive protein (Table 1), and negative rheumatoid factor (RF), negative anti-citrullinated C-peptide (anti-CCP) (Table 2), and myositis specific antibodies (MSA) were awaited. She also underwent bronchoscopy with bronchoalveolar lavage, with samples being sent for further studies, including cytology, which showed bronchial epithelial cells and squamous cells in the background of acute and chronic inflammation. A repeat CT scan showed chronic appearing peripheral interstitial disease with end-stage bibasilar honeycombing and superimposed airspace consolidation suggesting pneumonia as illustrated in Figure 2.

**Table 1:** Inflammatory marker trend.

	Initial	Repeat
Erythrocyte sedimentation rate (ESR) (0-3- mm/hr)	59	71
C-reactive protein (CRP) (<0.3 mg/L)	6.7	17.6

**Figure 3:**
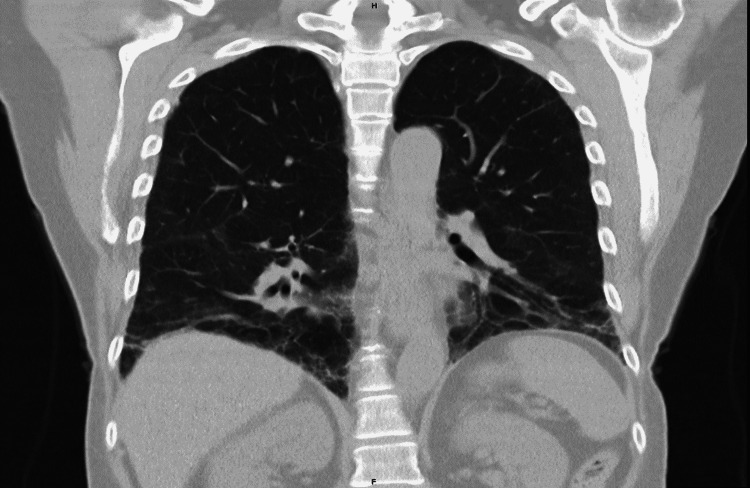
Basilar predominant interstitial/reticular pulmonary opacities, patchy ground-glass opacities suggesting NSIP. NSIP: Nonspecific interstitial pneumonia

The repeat laboratory results revealed elevated ESR and CRP (Table 1), and the awaited myositis-specific antibody was positive for anti-EJ antibody with a titer of >1:100 (Table 2). The patient was started on a pulse dose of methylprednisolone 1 gm for 3 days with to plan to taper over 3 months. Three months later, she underwent high-resolution CT, which revealed basilar predominant interstitial/reticular pulmonary opacities, patchy ground-glass opacity, and traction bronchiectasis compatible with ILD in a pattern suggesting NSIP (Figure 3). The patient recovered completely and significantly improved her exercise tolerance in a recent follow-up.

**Table 2:** Rheumatological tests result.

Tests	Result
Rheumatoid factor (RF)	Negative
Anti-citrullinated C-peptide (Anti-CCP)	Negative
Myositis specific antibody (MSA) (Anti-EJ antibody)	Positive Titer >1:100

## Discussion

This case illustrates the pulmonary manifestation as the initial manifestation of anti-ARS antibody syndrome without any extrapulmonary manifestation. The HRCT performed after the resolution of the acute symptoms revealed features suggesting NSIP. The initial diagnostic confusion raised in the era of the COVID-19 pneumonia epidemic despite persistent negative PCR results delayed the initiation of definitive treatment, which prolonged her hospital stay. This case also depicts the satisfactory response of pulse dose steroids in alleviating NSIP symptoms.

Idiopathic interstitial pneumonias can be classified as major, rare, and unclassified. Major categories include idiopathic pulmonary fibrosis, NSIP, cryptogenic organizing pneumonia, respiratory bronchiolitis interstitial lung disease, desquamative interstitial pneumonia, and acute interstitial pneumonia. The rare category includes idiopathic pleuroparenchymal fibroelastosis, lymphocytic interstitial pneumonia, and others. These lung pathologies usually manifest with other systemic conditions including infections, autoimmune diseases, toxins exposure, and so on. A clear understanding of the relationship with the triggering factors is not well described in the literature. As with other ILD, NSIP presents as progressive breathlessness with exertion and nonproductive cough. A high-resolution CT usually shows increased reticular markings, traction bronchiectasis, lobar volume loss, and ground-glass opacity predominantly in the lower lung fields.

Anti-EJ antibody is the least common antibody associated with NSIP and has mainly been reported in patients with dermatomyositis-associated ILD [7]. In a study of Chinese patients with anti-glycyl tRNA synthetase syndrome, ILD-related symptoms were the most common initial manifestations and most prevalent features [8]. In another study, the prevalence of NSIP reached 94.4% [9]. In a retrospective study conducted on 84 patients, an anti-EJ antibody was found in 14.29% of patients, and NSIP was the main type of clinical manifestation in the patients positive for anti-EJ antibody [6]. Taken together, these findings support the early consideration of anti-ARS antibodies when evaluating patients with clinical manifestations of ILD, which could prompt early diagnosis and appropriate treatment, potentially leading to better clinical outcomes. 

## Conclusions

Through this case, we would suggest clinicians that one should be cognizant of anti-ARS antibody syndrome during the evaluation of patients with ILD. The CT scan features suggesting NSIP should prompt an evaluation of anti-ARS antibodies, particularly the anti-EJ antibody, as NSIP is sensitive to steroid treatment. Early treatment with steroids showed favorable clinical outcome in our patient. Bigger studies are required to evaluate the types of anti-ARS antibodies and specific clinical manifestations to aid in early management and precisely predict the prognosis. 
